# ASSOCIATIVE AND EXECUTIVE INFLUENCES ON MEMORY FOR EVENTS AS REVEALED BY AGING AND DISTRACTION

**DOI:** 10.1093/geroni/igad104.2619

**Published:** 2023-12-21

**Authors:** Alan Kersten, Julie Earles, Megan Smithwick, Carlos Vaca Angus

**Affiliations:** Florida Atlantic University, Boca Raton, Florida, United States; Florida Atlantic University, Boca Raton, Florida, United States; Florida Atlantic University, Boca Raton, Florida, United States; Florida Atlantic University Wilkes Honors College, Boca Raton, Florida, United States

## Abstract

Diagnostic tests of memory functioning typically employ verbal material, potentially encouraging mnemonic strategies ranging from rehearsal to visual imagery. Poor performance could thus reflect either impairments in basic associative mechanisms or impairments in executive functions underlying strategy deployment. Kersten et al. (2018) developed a more ecologically valid test of episodic memory, the Person-Action Conjunction (PAC) Test, involving memory for who did what in events. They found that when many different actors performed the actions, memory for who performed each action was related to tests of associative functioning, whereas when only two actors performed all the actions, memory performance was related to executive functioning. The present research tested the ensuing prediction that manipulating the availability of executive resources through distraction should only impact performance when a small number of actors perform the actions. The PAC Test was administered to 207 young adults (M age = 20.39) and 163 healthy older adults (M age = 70.80), either with no distraction or while performing a tone counting distractor task. Young adults performed better than older adults at remembering who did what, consistent with an age-related associative deficit. When two actors performed all the actions, distraction impacted both age groups’ memory for which actor performed each action. When many different actors performed the actions, distraction had no specific impact on memory for who did what. Implications will be discussed for the development of diagnostic tests to differentiate impairments in basic memory mechanisms (e.g., early-stage Alzheimer’s disease) from impairments of executive functioning (e.g., vascular dementia).

